# Chronological Sequence of Leaf Phenology, Xylem and Phloem Formation and Sap Flow of *Quercus pubescens* from Abandoned Karst Grasslands

**DOI:** 10.3389/fpls.2017.00314

**Published:** 2017-03-06

**Authors:** Martina Lavrič, Klemen Eler, Mitja Ferlan, Dominik Vodnik, Jožica Gričar

**Affiliations:** ^1^Department of Yield and Silviculture, Slovenian Forestry InstituteLjubljana, Slovenia; ^2^Department of Agronomy, Biotechnical Faculty, University of LjubljanaLjubljana, Slovenia; ^3^Department of Forest Ecology, Slovenian Forestry InstituteLjubljana, Slovenia

**Keywords:** pubescent oak, cambium, radial growth, cell differentiation, leaf development, sub-Mediterranean

## Abstract

Intra-annual variations in leaf development, radial growth, including the phloem part, and sap flow have rarely been studied in deciduous trees from drought-prone environments. In order to understand better the chronological order and temporal course of these processes, we monitored leaf phenology, xylem and phloem formation and sap flow in *Quercus pubescens* from abandoned karst grasslands in Slovenia during the growing season of 2014. We found that the initial earlywood vessel formation started before bud opening at the beginning of April. Buds started to open in the second half of April and full leaf unfolding occurred by the end of May. LAI values increased correspondingly with leaf development. About 28% of xylem and 22% of phloem annual increment were formed by the time of bud break. Initial earlywood vessels were fully lignified and ready for water transport, indicating that they are essential to provide hydraulic conductivity for axial water flow during leaf development. Sap flow became active and increasing contemporarily with leaf development and LAI values. Similar early spring patterns of xylem sap flow and LAI denoted that water transport in oaks broadly followed canopy leaf area development. In the initial 3 weeks of radial growth, phloem growth preceded that of xylem, indicating its priority over xylem at the beginning of the growing season. This may be related to the fact that after bud break, the developing foliage is a very large sink for carbohydrates but, at the same time, represents a small transpirational area. Whether the interdependence of the chronological sequence of the studied processes is fixed in *Q. pubescens* needs to be confirmed with more data and several years of analyses, although the ‘correct sequence’ of processes is essential for synchronized plant performance and response to environmental stress.

## Introduction

Among all European regions, the Mediterranean appears to be the most vulnerable to climate change and subsequent increase in the frequency and intensity of extreme climatic events, such as drought and heat waves, which may create unprecedented climate-caused stress on trees and forest ecosystems (IPCC, 2014). In order to understand better the sensitivity and growth patterns of different tree species in natural adverse environments numerous studies have been carried out on this topic in recent years (e.g., Camarero et al., 2010; Nasr et al., 2011; Martinez del Castillo et al., 2016; Pérez-de-Lis et al., 2016b). Information about tree adaptation strategies and survival in marginal areas is crucial for our understanding of tree functioning and resilience, and is indispensable for future forest management (Tognetti et al., 2007; Hernandez-Santana et al., 2009).

Pubescent oak (*Quercus pubescens* Willd.), a deciduous ring-porous tree species, typically grows in a Mediterranean-type climate (Damesin and Rambal, 1995; Poyatos et al., 2005, 2007). It has developed various mechanisms and adaptations to survive in drought prone environments. Progressive stomatal closure, with a gradual and large decrease in water potential (Ψ) indicates its conservative water use. It decreases photosynthetic yield substantially during severe drought and shows efficient protection against high irradiance (Damesin and Rambal, 1995). Nevertheless, extreme drought events can lead to a severe decrease of water potential, loss of non-structural carbohydrates and loss of stem hydraulic conductivity, as shown by Nardini et al. (2016) for drought in the year 2012. Tognetti et al. (1998) studied the vulnerability of *Q. pubescens* to cavitation and estimated a water potential of -2.0 MPa as a threshold value causing 50% loss of water transport capacity (Ψ_50_). Such Ψ values can also be easily achieved in non-extreme dry summers (Nardini et al., 2016). This suggests that pubescent oak operates close to the point of xylem dysfunction. During summer, it protects against embolism by stomatal regulation which keeps water potentials above that causing hydraulic failure. Yet, stem conductivity can be affected also by frost-induced embolism, which first develops in large diameter vessels. According to Tyree and Cochard (1996) *Quercus* spp. suffer a high loss due to freezing and survival of the tree depends on the formation of a new ring of sapwood before leaf flush in spring.

In relation to xylem growth, the response of *Q. pubescens* to prolonged drought is reflected in reduced cambial cell production and, consequently, a lower proportion of latewood, as typical of ring-porous tree species (e.g., Panshin and de Zeeuw, 1980). Earlywood with, on the other hand, is more stable resulting in its smaller proportion in the case of wider xylem rings (Gričar et al., 2014). In addition, decreasing conduit size under drought, leading to a reduction in water-conducting capacity and a lower risk of cavitation, is a stress avoidance strategy of *Q. pubescens* (Eilmann et al., 2006; Zweifel et al., 2006). However, a reduction in the number of narrow latewood vessels might, in turn, cause its higher susceptibility to drought (Eilmann et al., 2009). In Slovenia, *Q. pubescens* is one of the prevailing tree species in the Karst region (Ferlan et al., 2016) and is ecologically important since it grows in forests that prevent degradation of vulnerable, shallow and erosion-prone soil (Brus, 2004). Information about its growth and survival strategies in this region therefore deserves more attention.

The radial stem growth of a tree is driven by the water status, which controls the metabolism of the entire tree, and carbon status, as a source of compounds for cambial activity and cell differentiation (Hsiao and Acevedo, 1974; Zweifel et al., 2006; Steppe et al., 2015). Due to their tight coupling, any changes in water-carbon relations (Zweifel et al., 2006) have an impact on xylem and phloem formation processes, affecting their ratios and specific structure (Hinckley and Lassoie, 1981; Gricar et al., 2015; Jyske and Hölttä, 2015; Sass-Klaassen et al., 2016). In this respect, intra-annual xylem and phloem formation analyses are crucial for a better understanding of the mechanisms underlying the environmental response of the xylem and phloem anatomy of different tree species. Information on intra-annual xylem growth of deciduous tree species growing in unfavorable growing conditions, such as Mediterranean areas, is scarce (González-González et al., 2013; Pérez-de-Lis et al., 2016a,b) although populations growing in such areas, together with rear-edge populations, are particularly under threat due to anticipated climate change (IPCC, 2014). Phloem observations in trees from sub-Mediterranean areas are even more rare and have been mainly performed on conifers (Gričar et al., 2016).

Intra-annual radial growth analyses are often coupled to leaf phenological observations to assess their linkage to photosynthesis and water transport (e.g., Michelot et al., 2012; Prislan et al., 2013; Copini et al., 2016; Kitin and Funada, 2016; Sass-Klaassen et al., 2016), since new photoassimilates transported in phloem sap are substrates used for radial growth increment (González-González et al., 2013). The relationship between these two developmental processes is therefore especially relevant in cambial reactivation, because expanding leaves are the main sources of auxin, which has a major role in cambial activity and cell expansion (Lachaud et al., 1999; Aloni, 2013). In *Q. pubescens*, xylem growth starts before bud burst and almost half of the xylem increment is formed at the time of leaf unfolding (Zweifel et al., 2006). Because of tylose formation in the major part of earlywood vessels of the previous xylem increments (Bréda and Granier, 1996), newly formed earlywood vessels are therefore essential to provide a water supply to the crown in a ring-porous *Q. pubescens* (Zweifel et al., 2006).

Since studies on leaf phenology, intra-annual radial growth and water relations in trees generally exclude the phloem part, we investigated these key processes (i.e., leaf phenology, xylem and phloem formation and sap flow) jointly in *Q. pubescens* trees from abandoned karst grasslands in Slovenia in 2014. In particular, three main aims were addressed: (1) to define the main milestones in the formation of xylem and phloem annual increments; (2) to compare the time course and interdependance of leaf development, phloem formation, xylem formation and sap flow measurements, with special attention to the beginning of the growing season and (3) to evaluate the obtained tree-anatomical and eco-physiological measurements in relation to local environmental conditions.

## Materials and Methods

### Study Site Characteristics

The study site was located in karst grassland at Podgorski Kras (45°32′56.3″N, 13°54′36.1″E, 429.4 m a.s.l.) in Slovenia, which was abandoned ca. 30 years ago from agricultural use after centuries of low intensity sheep and cattle grazing. Abandonment lead to encroachment of various woody plant species, among which pubescent oak dominates, growing in patches or solitary with an average height of 7 m and average age ca. 30–40 years.

The site is influenced by a sub-Mediterranean climate, characterized by fairly harsh winters, and dry and hot summers when drought periods frequently occur. The average annual temperature in the period 1981–2010 was 10.4°C, the average daily maximum 16.5°C and the average daily minimum 5.4°C. The lowest average temperature was recorded in January (1.3°C) and the highest in July (20.1°C). The average total annual precipitation was 1170 mm, with two annual rainfall peaks; in autumn and in late spring. Meteorological data for the region were obtained from the nearby Ilirska Bistrica climate station belonging to the Slovenian Environment Agency (ARSO).

The dominant soil type is rendzic with very uneven soil depth and rocky surface. The soil has mainly a clay texture with a low amount of plant nutrients, 12–15% proportion of organic matter in the top layer and pH value 6.9. Severe weather conditions, shallow soil and frequent wind diminish the impact of high precipitation, so water stress frequently occurs in the growing season (March–October) (Ferlan et al., 2011).

### Weather Conditions in 2014

**FIGURE 1:**
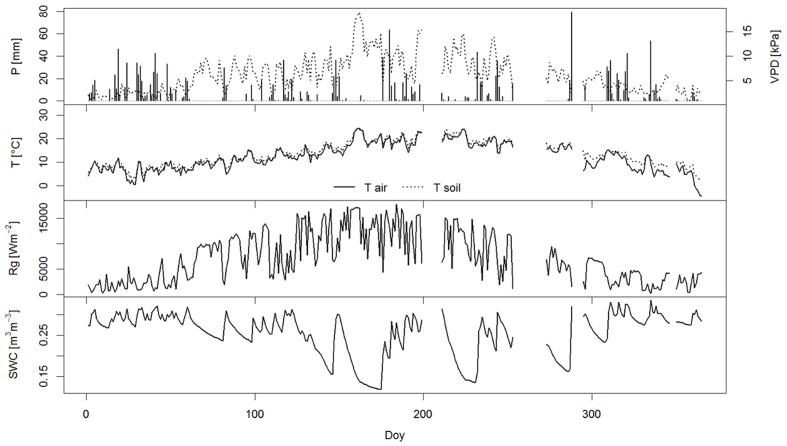
**Measured meteorological parameters at the study site Podgorski Kras in 2014, including daily sum of precipitation (P), daily sum of vapor pressure deficit (VPD), mean daily air and soil temperature (T), daily sum of sum solar radiation (Rg) and mean daily soil water content (SWC)**.

In 2014, daily data of meteorological parameters (air and soil) temperature (T), precipitation (P), vapor pressure deficit (VPD), solar radiation (Rg) and soil water content (SWC) measured at half-hourly intervals were obtained from a micrometeorological tower in the close vicinity of the research plot (Ferlan et al., 2011). The year 2014 was unusually wet, exceeding the long-term average of sum of precipitation by about 50% (**Table 1** and **Figure 1**). A more detailed description of the measured environmental parameters at the study site in 2014 is shown in **Table 1**.

**Table 1:** Detailed description of the measured environmental parameters at the study site in 2014.

Precipitation (mm)	Yearly average	1746
	Wettest months (January, February, November)	253
	Driest months (March, September)	67
Air temperature (°C)	Yearly average	12.1
	Average daily maximum	24.2
	Average daily minimum	-4.5
	Annual amplitude	38.7
	Daily amplitude	20.6
	Average in warmest months (June–August)	19.3
	Absolute maximum	32.2
	Average in coldest months (December–February)	5.8
	Absolute minimum	-6.4
Soil temperature (°C)	Yearly average	13.5
	Daily maximum in June	31.9
	Daily minimum in January	-0.5
VPD (kPa)	Yearly average	6.1
	Average daily maximum	19.0
	Average daily minimum	0.9
	Monthly maximum (June)	33.8
	Monthly minimum (January)	0.6
Rg (Wm-2)	Average annual	0.25
	Average during vegetation period	0.23
	Average during non-growing season	0.28
SWC (m3m-3)	Average annual	150
	Average during vegetation period	224
	Average during non-growing season	84


### Tree Selection and Leaf Phenological Observations

At the beginning of the 2014 growing season, we randomly selected six solitary healthy pubescent oaks (*Quercus pubescens* Willd.) without any visible injuries on stems and roots. The trees were 35 ± 5 years old and 7 ± 0.5 m high, with diameters at breast height (DBH) of 0.18 ± 0.015 m. The selected oaks were equipped for sap flow measurements and intra-annual microcore sampling was performed in the period March–October of 2014.

Leaf phenological observations and leaf area index (LAI) measurements were carried out on all oaks in the first part of the growing season, i.e., March–July. On the northern side of each tree, one branch was selected for phenological observations. We focused on phenological phase leaf unfolding, which was divided into five subclasses: 1 – buds are fully closed, 2 – buds are open (leaf appearance), 3 – the beginning of leaf unfolding (33–66% leaf development), 4 – leaf unfolding throughout the canopy (66–99% leaf development), 5 – full leaf unfolding. We took weekly images of a selected part in the crown with a digital camera to document leaf development.

### Leaf Area Index Measurements

In the period March–July, we measured the LAI, i.e., one-sided leaf surface area per ground surface area (m^2^m^-2^) with a LAI-2200 Plant Canopy Analyzer (LI-COR Inc., USA). We performed measurements at weekly intervals following the protocol for measuring LAI of isolated trees (LAI-2200, 2012). At each tree, four “below canopy” readings were taken in N, E, S, and W orientations, placing the sensor equipped with a 270° view restrictor next to the trunk. Data of gap fraction measurements were analyzed with FV2200 software, version 2.0.0 (LI-COR Inc., USA) in which the average LAI values for each tree were calculated.

### Sap Flow Measurements

At the beginning of the growing season (i.e., first decade of April), the selected oaks were equipped with SFM1 Sap Flow Meters based on the Heat Ratio Method (SFM1 instrument, ICT International, Australia). According to the protocol described in Burgess and Downey (2014), SFM1 were installed on the north side of the tree, 1 ± 0.1 m above the ground, with a 0.5 mm distance between the temperature probes and the heater. Data were stored continuously at 10-min intervals in binary notation and monthly downloaded in Sap Flow Tool software (ICT International, 2014). In the software, data were analyzed using correction factors (e.g., stem diameter, bark thickness, and sapwood depth), which were acquired at the end of the measurement campaign (Pfautsch et al., 2010; Burgess and Downey, 2014). The average sapwood width of pubescent oaks was 2.13 ± 0.6 cm, so only data of the outer temperature thermistor located in the conducting wood were applied. The inner temperature thermistor was already in hardwood, in which water transport is interrupted due to the presence of tyloses in the large earlywood vessels (Cochard and Tyree, 1990). These data were not therefore used for analyses. For determination of the actual sap flux density, zero flow values were calculated for each tree from the data obtained from the period before leaf unfolding, i.e., DOY 1–112 (Do and Rocheteau, 2002; Vandegehuchte and Steppe, 2013). Due to power supply problems, a few gaps in SF data occurred (12% data loss) (**Figure 1**).

### Microcore Collection, Section Preparation and Tissue Analyses

Microcores were collected at weekly intervals from March until August of 2014, using a Trephor tool (Rossi et al., 2006). Additionally, microcores were collected in mid-October, when phloem and xylem rings were fully developed. The samples were collected at 0.7–1.7 m above the ground following a spiral up the stem. In order to avoid wound effects, sampling locations were separated by 5 cm. Each microcore contained phloem (non-collapsed and collapsed), cambium and at least two of the last-formed xylem rings. Immediately after removal from the trees, the samples were fixed in ethanol-formalin acetic acid solution (FAA) (Gričar et al., 2007). After 1 week, the samples were dehydrated in a graded series of ethanol, infiltrated with D-limonene (Bio Clear, Bio Optica, Milano, Italy) and embedded in paraffin blocks (Paraplast plus, ROTH, Karlsruhe, Germany) (Rossi et al., 2006). Transverse sections of 8–12 μm thickness were cut with a Leica RM 2245 rotary microtome (Leica Microsystems, Wetzlar, Germany). The sections were stained with a safranin (Merck, Darmstadt, Germany) (0.04%) and astra blue (Sigma–Aldrich, Steinheim, Germany) (0.15%) water mixture (van der Werf et al., 2007) and mounted in Euparal (Waldeck, Münster, Germany). They were observed under an Olympus BX51 light microscope (Tokyo, Japan) using transmission and polarized light modes. Histometrical analyses (i.e., determination of phenological phases of cambial activity and cell differentiation, and increment measurements) were performed with the Nikon NIS-Elements Basic Research v.2.3 image analysis system (Tokyo, Japan).

On the sections, the number of cell layers in the cambium was counted, as well as the widths of currently formed xylem, and phloem increments along three radial files were measured and then averaged. Additionally, the following phenological phases of cambial activity were assessed: (1) onset of cambial cell production, (2) maximum rate of xylem and phloem cell production, (3) cessation of cambial cell production, (4) cessation of the differentiation process in terminal latewood tracheids and (5) transition from earlywood to latewood and from early to late phloem. Phases were assessed for each tree and were computed in days of the year (DOY).

The phenological phases of cambial activity and cell differentiation listed above were identified and interpreted within the context of the multi-seriate concept that the vascular cambium comprises both the cambial initial cells and the xylem and phloem mother cells (Plomion et al., 2001). The observed xylem and wood formation phases were defined as follows: (1) the onset of cambial cell production was identified as an increased number of thin-walled cambial derivatives (Prislan et al., 2011); (2) the maximum rate of new xylem and phloem cell production was calculated and plotted using a Gompertz function that indicated the maximum growth rate at the inflection point of the curve (Rossi et al., 2003); (3) cessation of cambial cell production was identified as the time at which no new thin-walled cells were observed adjacent to the cambium and the number of cambial cells was comparable to the number before its reactivation in spring; (4) cessation of xylem differentiation was identified by the complete lignification of the terminal latewood cells, as indicated by cell walls stained completely red by the safranin-astra blue procedure; (5) transition from earlywood to latewood was determined when small latewood vessels were no longer arranged in rings, which is characteristic of large earlywood vessels (Gričar, 2010); (6) transition from early to late phloem was identified by the appearance of small, tangentially orientated groups of phloem fibers separating the two parts (Srivastava, 1964; Gričar, 2010).

### Data Analysis

Analyses were performed using the statistical software R, version 3.2.3 (R Core Team, 2015). Data were displayed as time-series and analyzed with descriptive statistics. The widths of xylem and phloem increments were fitted to the Gompertz function (Rossi et al., 2003) using the nlme package in R. Model fits were evaluated by computing pseudo-R2 by comparing residual variance of the full model against the residual variance of an intercept-only null model. The first derivative of the Gompertz function was calculated to determine maximum cambial cell production of xylem and phloem rings during the growing season.

## Results

### Leaf Phenology and LAI Measurements

Leaf phenological observations were performed from March until July, with the main focus on early spring leaf development. Typical of many *Quercus* sp. are marcescent leaves that are dead and retained through the winter (Sánchez de Dios et al., 2009); consequently, there was still a small proportion of old leaves from the previous year attached to the branches of the pubescent oak in early spring, possibly increasing our LAI values. Buds were fully closed until the second half of April, when they started to open (DOY 110.5 ± 5.5), followed by the appearance of the first leaves. By the end of April, half of the trees’ canopy was covered with leaves. Full leaf unfolding occurred by mid-May (DOY 131.5 ± 5.5). Thus, in 2014 the period from bud opening to full leaf unfolding lasted on average 21 days (**Figure 3**). LAI values increased correspondingly with leaf development. When the first leaves appeared in mid-April, LAI values were 0.27 ± 0.1 m^2^m^-2^ and reached an average maximum of 2.02 m^2^m^-2^ at the beginning of June, 3 weeks after full leaf development. Maximum LAI values varied among the trees; for example, in three individuals LAI was 3.45 ± 0.4 m^2^m^-2^, while in one oak only 0.86 m^2^m^-2^.

### Seasonal Dynamics of Xylem and Phloem Formation

**FIGURE 2:**
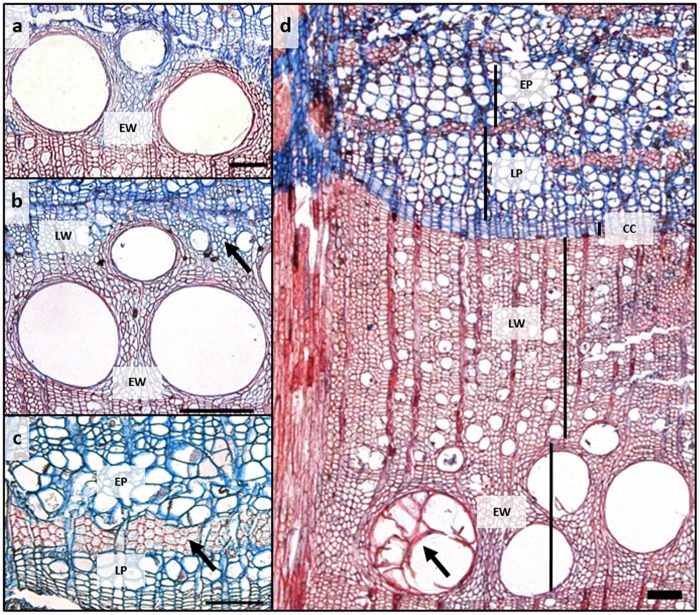
**(a)** Fully developed first ring of earlywood vessels; **(b)** Transition from early to latewood (black arrow); **(c)** Transition from early to late phloem (black arrow) and **(d)** Fully formed xylem and phloem increments in *Quercus pubescens* in 2014. Black arrow denotes earlywood vessel filled with tyloses. CC, cambium; EW, earlywood; LW, latewood; EP, early phloem; LP, late phloem, Bars = 100 μm.

**FIGURE 3:**
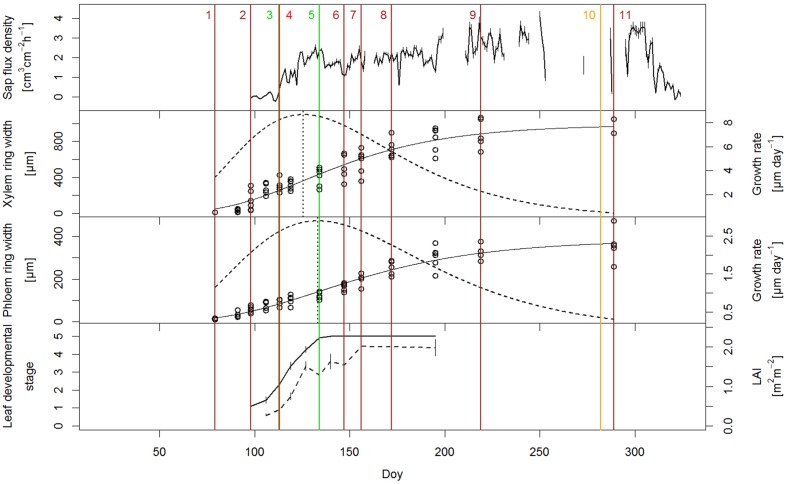
**Relationship between various measured parameters (sap flow density, seasonal dynamics of xylem and phloem growth ring formation and leaf development) in *Quercus pubescens* during the growing season of 2014.** Xylem and phloem development are presented as cumulative growth (solid line) and as weekly increment growth (dashed line). Leaf development is presented as phenological stages (solid line) and LAI measurements (dashed line). The most important milestones of the analyzed parameters are indicated with numbers: (1) onset of cambial cell production (DOY 79); (2) onset of expansion of initial earlywood vessels (DOY 98); (3) full bud opening (leaf appearance) (DOY 113); (4) complete development of initial earlywood vessels (DOY 113); (5) full leaf unfolding (DOY 134); (6) transition from earlywood to latewood (DOY 147); (7) transition from early phloem to late phloem and reaching peak of LAI (DOY 156); (8) complete development of earlywood (DOY 172); (9) end of cambial cell production (DOY 219); (10) autumn leaf senescence (DOY 282), and (11) cessation of xylem growth ring formation (DOY 289).

In the third week of March, when we started the microcore sampling, cell division in the cambium had just started, as inferred from the increased number of cambial cells (7–8 cell layers) compared to that of the dormant cambium (4–5 cell layers) at the end of the growing season in October. In addition, in some of the trees, initial sieve tubes of early phloem started to expand adjacent to the cambium (16.1 ± 3.8 μm). In the first 3 weeks of radial growth, phloem growth exceeded that of xylem but from 8 April until the cessation of cambial cell production, xylem growth surpassed that of phloem. By the third week of June, earlywood vessels were fully created (**Figure 2a**). The average duration of the earlywood vessel enlargement period was 46 ± 4 days and of earlywood vessel formation 89 ± 11 days. Latewood started to form in the third week of May (**Figure 2b**), while the entire earlywood part was fully formed in the second half of June. In 2014, *Q. pubescens* generally developed one to two rows of earlywood vessels, with the largest ones being mostly located in the first row, at the growth ring boundary (**Figure 2b**). The transition from early to late phloem occurred at the end of May, as evident from the formation of the groups’ phloem fibers (**Figure 2c**). The peak of cambial production of xylem cells occurred at the beginning of May (DOY 125.6), when on average 8.7 μm of xylem tissue was formed per day, as calculated from the first derivative of the Gompertz function (**Figure 3**). At that time, 25% of the annual xylem increment was formed. For phloem, maximum cambial cell production (2.9 μm per day) took place 2 weeks later than in the case of xylem. At that time, 40% of the annual phloem increment was formed. At the height of cambial cell production, the cambium was more than 10 cell layers wide, with the number gradually decreasing thereafter. At the beginning of August, cambial cell production stopped simultaneously on both sides. In 2014, the average width of the completed xylem increment was 1324 ± 440 μm (min = 889 μm; max = 2311 μm) and of phloem 337 ± 69 μm (min = 230 μm; max = 482 μm) (**Figure 2d**). Earlywood and early phloem parts occupied 36.8 and 40.5%, respectively, of the annual growth increments.

### Sap Flow

Before bud break, we observed minimum sap flux density, which reached a daily average of 0.012 cm^3^cm^-2^h^-1^. Sap flux density and leaf unfolding showed a linear relationship in the period from milestone (3) to (5), as illustrated in **Figure 3**, whereas later, in the late stage of leaf development, a decrease of sap-flow was observed due to reduced transpirational demand (rain period).

The average sap flux density in the summer season when the canopy was fully developed but non-senescent (from DOY 134 to 280) was 2.19 cm^3^cm^-2^h^-1^. Fairly high fluctuations were found. These variations could largely be explained by precipitation, soil water availability and fluctuating water vapor pressure deficit. Previous research at the same site has shown that ecosystem evapotranspiration was severely reduced at a SWC lower than 0.145 m^3^m^-3^, which indicates a reduction of stomatal conductivity at the stand level (Ferlan et al., 2011). When sap-flow data of *Q. pubescens* were filtered for days with higher SWC, 46.2 and 37.0% of the variability of sap-flow density could be explained by water vapor deficit and Rg, respectively. The highest mean value of sap flux density was measured at the end of August: 8.65 cm^3^cm^-2^h^-1^. High densities persisted in part of November and then decreased in December, reaching early spring values at the end of the year. The drop in values coincided with the mass autumnal brown coloring of the leaves on trees.

### Time Course of Leaf Development, sap Flow and Seasonal Radial Stem Growth in the First Part of the Growing Season

The expansion of the initial earlywood vessels already started at the beginning of April, when buds were still closed (**Figure 3**). At that time, no sap flow was recorded; its value was below 1 cm^3^cm^-2^h^-1^. By mid-April, when the buds were still closed, about 28% of the xylem and 22% of phloem annual increment were formed. In the last decade of April, buds started to open and the first leaves appeared. At the same time, initial earlywood vessels were fully lignified and thus ready for water transport. Sap flow became active and was contemporarily increasing with leaf development and LAI values. In the meantime, about one third of the xylem and one fourth of the phloem annual increments were created. Full leaf unfolding occurred in mid-May, when about 40% and 33% of xylem and phloem increments, respectively, had formed. In the period of leaf development (DOY 113–134) average sap flux density was 1.60 cm^3^cm^-2^h^-1^. Transition from earlywood to latewood occurred 2 weeks after full leaf development. Transition from early to late phloem, which took place a week later, coincided with the end of LAI increase.

## Discussion

### Synchronicity of Leaf Development and Increase of LAI Values

The increase in LAI values in the period from mid-April till mid-May corresponded to phenophases of leaf unfolding Furthermore, LAI values continued to rise for about 3 weeks after full leaf development, indicating canopy extension and the development of new shoots (height growth). Average LAI values of our *Q. pubescens* trees were lower (2.2 m^2^m^-2^) than of some other *Quercus* sp. from the literature, ranging from 3 to 7 m^2^m^-2^ (Bréda and Granier, 1996; Jonard et al., 2011; Burner et al., 2014). However, our measurements were in line with LAI values of *Quercus robur* stands (2.26 m^2^m^-2^) (Bequet et al., 2012). Since LAI is an indicator of soil fertility and/or stand productivity (Bréda and Granier, 1996) and is spatially highly variable (Bequet et al., 2012), differences in all values in different studies can be attributed to all these factors, as well as to differences in tree density, because our study was carried out on solitary oaks. Finally, environmental conditions also highly influence leaf development and LAI values (Mooney, 1972).

### Seasonal Dynamics of Xylem and Phloem Formation

The main growth period (i.e., leaf expansion and phloem and xylem growth) was April–June, which is very similar to other species (e.g., González-González et al., 2013; Prislan et al., 2013). After that period, carbon is still assimilated, partly used for secondary wall biosynthesis but mostly for below-ground growth (Barbaroux et al., 2003) or storage reserves (Hoch et al., 2003). The transition from early to late phloem occurred after transition from earlywood to latewood. The transition from early to late phloem is associated with changes in photosynthetic allocation. Early in the growing season, a large proportion of the photosynthates is used for respiration, cell division and differentiation, and biosynthesis. As the rate of cell division slows, the principal sink for photosynthate shifts to storage in wood parenchyma, both above and below ground (Taiz and Zeiger, 2006). In addition, the second part of the growing season is important for storage of carbohydrate resources (in parenchyma) (Barbaroux and Bréda, 2002).

The phloem increment represented one fourth of the annual radial increment in 2014 (1.7 ± 0.5 mm). The xylem increment thus predominated, which is typical of productive oaks having a xylem ring wider than 0.75 mm (Gričar et al., 2014). Xylem and phloem ring widths, as well as proportions between xylem and phloem increments, and early and late parts of xylem and phloem of *Q. pubescens*, are comparable to measurements in *Q. robur* from lowland forests, suggesting that they might be general patterns for ring-porous oaks (Gričar et al., 2014). Under drought, *Q. pubescens* mainly reduces the latewood proportion, whereas earlywood remains more or less constant (Eilmann et al., 2009). Large earlywood vessels are most important in terms of water-conductivity, since the bulk water transport in oak occurs in earlywood of the youngest xylem rings (Ellmore and Ewers, 1985). Each spring, therefore, at least one new tangential row of earlywood vessels is formed in order to regenerate its maximum conductivity every spring, which is a successful strategy in summer-dry climates (Eilmann et al., 2009). Small vessels and tracheids in latewood, on the other hand, which are formed in the summer months, are also important for water transport because they represent a safety component of the xylem in ring-porous species. Their role is negligible when conditions are favorable but is critical in the case of cavitation of earlywood vessels (Granier et al., 1994).

### Linkage between Leaf Unfolding and Radial Growth

The timing of vessel formation and leaf phenology differs among *Quercus* sp. (Suzuki et al., 1996; Sass-Klaassen et al., 2011; Takahashi et al., 2013), especially when comparing temperate and sub-Mediterranean oaks (González-González et al., 2013; Pérez-de-Lis et al., 2016b). We confirmed the observations of Zweifel et al. (2006) that a substantial amount of current xylem increment in *Q. pubescens* is formed at the time of bud burst and full leaf unfolding (28 and 40%, respectively). Since tyloses in large earlywood vessels of the previous growth rings impede water transport in ring-porous *Q. pubescens* (Cochard and Tyree, 1990), hydraulic conductivity is strongly reduced in early spring (Bréda and Granier, 1996). Initial earlywood vessels formed before bud break are therefore essential in order to provide a water supply to the young transpiring leaves in the crown (Zweifel et al., 2006). This pattern demonstrates that xylem tissue formed before full leaf development relies on carbohydrates stored during the previous growing season, particularly during the summer and autumn of the previous year (Barbaroux and Bréda, 2002).

With few exceptions (e.g., Prislan et al., 2013), the phloem part has been largely overlooked in studies on leaf phenology and intra-annual radial growth, despite its main role in the transport of carbohydrates from photosynthetic and storage tissues (source) to areas of active growth and metabolism (sinks) (Oparka and Robert, 1999). We found that at the time of bud burst and full leaf unfolding, 22 and 33%, respectively, of the early phloem part was formed, mainly consisting of large sieve tubes. In addition, in the initial 3 weeks of radial growth, phloem growth preceded xylem growth, as already previously observed in *Q. petraea* (Gričar, 2010). Rapid production of phloem indicates its priority over xylem at the beginning of the growing season, which may be related to the fact that, shortly after the beginning of leaf development, the developing foliage is a very large sink for carbohydrates but, at the same time, represents a small transpirational area. It can also be presumed that the hydraulic conductivity and water storage of sapwood developed in preceding years efficiently support the water balance of leaves at this, very early stage of development. On the other hand, one might expect that existing phloem connections might limit carbohydrate allocation at the very start of the season. Sieve tubes function for only one to two growing seasons and late phloem cells may remain functional until new phloem is produced in spring (Zamski and Zimmermann, 1979). Furthermore, overwintering undifferentiated cambial derivatives may differentiate into sieve elements before new elements are formed by the dividing cambium (e.g., Derr and Evert, 1967). Thus, newly formed phloem cells are important to link the storage tissues to developing leaves, which require photosynthates for respiration and biosynthesis in spring (Barbaroux et al., 2003).

Not much is known about the phloem formation patterns of deciduous trees in sub-Mediterranean areas, especially species with marcescent leaves. These leaves are presumed to be dead and retained on a tree through the winter (Sánchez de Dios et al., 2009). Abadía et al. (1996) demonstrated that marcescent leaves from the upper part of the tree crown of *Q. subpyrenaica*, unlike senescent leaves from the lower part of the crown, are capable of some photosynthetic activity in the last 1–2 months of the growing season (September and October), which significantly increases the possibility of carbon assimilation during the period when high light intensities and mild temperatures may still occur. Whether marcescent behavior of *Q. pubescens* affects carbohydrate reserves and thus also radial growth in the spring of the following growing season remains to be examined.

### Sap Flow With Respect to Leaf Phenology and Environmental Conditions

Early spring patterns of xylem sap flow and LAI were similar, indicating that water transport in the oaks broadly followed canopy leaf area development. A similar relation was found for *Quercus prinus* by Wullschleger et al. (2001). Sap flow is in general very well correlated with leaf area, with an almost linear relationship (Vertessy et al., 1995). After the full development of foliage, transpiration and sap-flow are mainly governed by climatic/weather (precipitation) variability, causing changes in SWC, vapor pressure deficit and light. A study by Wullschleger et al. (2001) showed that, within the season, 85% of daily sap flow variability in *Q. prinus* can be explained by radiation, vapor pressure deficit and fractional LAI. Meteorological network data and our measurements of environmental parameters in the field (**Figure 1**) showed that 2014 was atypically wet. The sum of precipitation and number of rainy days in summer exceeded the 10-year average by 35 and 33%, respectively. Sharp decreases of sap flux density during the season can therefore be attributed to reduced transpirational demand and low light conditions during wet days. During a short dry and hot early summer period, in mid-June SWC decreased below SWC_critical_ (0.145 m^3^m^-3^, Ferlan et al., 2016) and sap flux density reached only medium values (1.89 cm^3^cm^-2^h^-1^), despite the Rg being high. At the same time, (June 21) leaf water potential measurements revealed a Ψ_midday_ value of -2.67 ± 0.13 MPa (Vodnik et al., unpublished data). It can be concluded that the transpiration flow of the pubescent oak was reduced by substantial stomatal closure induced by low water potential and high VPD. This can be supported by the study of Nardini et al. (2016), who report that *Q. pubescens* leaf conductance to water vapor decreased by 85% when comparing a period with ample of water (Ψ_midday_ = -1.5 MPa) and an extremely dry period (Ψ_midday_ = -3.5 MPa).

### Is the Chronologial Sequence of the Studied Patterns (Leaf Development, LAI, Radial Growth and Sap Flow) Fixed in *Q. pubescens*?

Understanding tree phenology, growth patterns and physiology is crucial to better understanding how trees cope with environmental changes (Granier et al., 1994; Sass-Klaassen et al., 2016). With *Q. pubescens*, a substantial amount of current xylem increment is formed before leaf unfolding, in order to provide essential hydraulic conductivity for axial water flow during leaf development, which is in line with the findings of Zweifel et al. (2006) for the same species. Comparing intra-annual xylem and phloem formation with other data (Gričar, 2010; Gričar et al., 2014) suggests that these might be general radial growth patterns for ring-porous oaks.

However, based on only 1-year of observation it is too speculative to conclude the interdependence of the chronological sequence of the studied processes (leaf development, LAI, xylem and phloem formation and sap flow) in *Q. pubescens*. Data of several growing seasons, including years with extreme events typical for this site (e.g., drought, fires) will reveal to what extent the time course of the studied variables is fixed and species-specific and how much it is influenced by environmental factors. After all, the ‘correct sequence’ of development of leaf unfolding, phloem and xylem growth and others, such as root growth, are essential for synchronized plant performance and response to environmental stress.

## Author Contributions

Conception or design of the work: DV, MF, and KE; Data collection: ML, DV, and KE; Data analysis and interpretation: all authors; Drafting the article: JG, ML, DV, and KE; Critical revision of the article: all authors; Final approval of the version to be published: all authors.

## Conflict of Interest Statement

The authors declare that the research was conducted in the absence of any commercial or financial relationships that could be construed as a potential conflict of interest.
